# Audio-Visual and Meaningful Semantic Context Enhancements in Older and Younger Adults

**DOI:** 10.1371/journal.pone.0152773

**Published:** 2016-03-31

**Authors:** Kirsten E. Smayda, Kristin J. Van Engen, W. Todd Maddox, Bharath Chandrasekaran

**Affiliations:** 1 Department of Psychology, The University of Texas at Austin, Austin, Texas, United States of America; 2 Department of Psychological and Brain Sciences, Washington University in St. Louis, St. Louis, Missouri, United States of America; 3 Communication Sciences and Disorders Department, The University of Texas at Austin, Austin, Texas, United States of America; Birkbeck College, UNITED KINGDOM

## Abstract

Speech perception is critical to everyday life. Oftentimes noise can degrade a speech signal; however, because of the cues available to the listener, such as visual and semantic cues, noise rarely prevents conversations from continuing. The interaction of visual and semantic cues in aiding speech perception has been studied in young adults, but the extent to which these two cues interact for older adults has not been studied. To investigate the effect of visual and semantic cues on speech perception in older and younger adults, we recruited forty-five young adults (ages 18–35) and thirty-three older adults (ages 60–90) to participate in a speech perception task. Participants were presented with semantically meaningful and anomalous sentences in audio-only and audio-visual conditions. We hypothesized that young adults would outperform older adults across SNRs, modalities, and semantic contexts. In addition, we hypothesized that both young and older adults would receive a greater benefit from a semantically meaningful context in the audio-visual relative to audio-only modality. We predicted that young adults would receive greater visual benefit in semantically meaningful contexts relative to anomalous contexts. However, we predicted that older adults could receive a greater visual benefit in *either* semantically meaningful or anomalous contexts. Results suggested that in the most supportive context, that is, semantically meaningful sentences presented in the audiovisual modality, older adults performed similarly to young adults. In addition, both groups received the same amount of visual and meaningful benefit. Lastly, across groups, a semantically meaningful context provided more benefit in the audio-visual modality relative to the audio-only modality, and the presence of visual cues provided more benefit in semantically meaningful contexts relative to anomalous contexts. These results suggest that older adults can perceive speech as well as younger adults when both semantic and visual cues are available to the listener.

## Introduction

Perhaps the most critical feature of the complex auditory soundscape in which we live is the speech of the people around us. Although background noise often degrades the speech signal, noise rarely disrupts ongoing conversations. This is partly due to various contextual cues listeners use to mitigate the deleterious effects of background noise. Of particular importance in such situations are visual [1–5] and semantic [5–8] cues. While there are other cues listeners may use to understand speech in challenging situations, the modulatory influences of visual cues and semantic context are well-studied in young adults and known to be important for speech perception in noise [4–6]. Prior research in young adults suggests that individual differences in speech perception in noise can be partly explained by differential use of visual cues and semantic context. In the present study, we investigate the interaction of these two cues in enhancing speech perception across age groups.

As we age, our ability to hear declines. Lin and colleagues found that 63.1% of adults aged 70 years and older in the United States experience some form of hearing loss, as defined by the World Health Organization [9]. Even so, older adults, for the most part, maintain their ability to understand speech. This is partly due to increased reliance on non-auditory cues. A large body of research suggests that older adults receive the same amount of visual benefit as younger adults during speech perception [1–3,10]. For instance, older adults and younger adults showed similar amounts of visual benefit when asked to repeat phonemes in multi-talker babble across a wide range of signal-to-noise ratios (SNRs; Power_signal_-Power_noise_ in decibels; [10]). Older adults also showed similar amounts of visual benefit to young adults across SNRs during a word identification task, except at the most difficult SNR (-16 dB), where older adults received less visual benefit than young adults [10]. Older adults also received the same amount of visual benefit as middle-aged adults when asked to repeat sentences presented audio-visually in speech envelope noise [11]. Finally, Sommers, Tye-Murray and Spehar (2005), found that older adults and young adults used visual cues to the same extent when asked to identify consonants, words, and semantically meaningful sentences using audio-visual presentation in 20-talker babble noise when accuracy during the audio-only presentation was controlled for between age groups [3].

Relative to visual cues, results are mixed as to whether or not older adults make greater use of semantic context compared to younger adults. Some studies have found that young and normal-hearing older adults receive the same amount of performance boost in identifying final words of sentences in highly predictive semantic contexts relative to low-predictability contexts. This has been found in both multi-talker babble [12] and spectrally-shaped noise [13]. Pichora-Fuller and colleagues, on the other hand, found that older adults (with normal hearing or with hearing loss) benefitted more than younger adults from a high degree of semantic context when identifying sentence-final words in multi-talker babble [7]. Older adults’ use of semantic cues to aid in speech perception also appears to make them more susceptible than young adults to false hearing: when a sentence is highly predictive of a particular final word but a non-predicted word is presented, older adults will report high confidence in their incorrect (but semantically predicted) responses [14].

In the previous sections we discussed the role of visual and semantic cues in independently modulating speech intelligibility. A recent study conducted on younger adults shows that these cues interact in enhancing speech intelligibility. Van Engen and colleagues (2014) found that young adults received greater visual benefit when there was a strong semantic context relative to a weak semantic context during a speech-in-noise perception task [5]. One possible explanation for their findings is that visual cues can be used more effectively when the set of possible words has already been significantly constrained by semantic context. Critically, the extent to which the semantic context may increase the visual benefit garnered during speech perception has never been studied in older adults. Given the literature suggesting that older adults may rely more strongly on semantic context than younger adults, it is important to examine whether semantic context modulates older adults’ ability to use visual cues during speech perception.

To explore these issues, we presented older adults (ages 60–90) and young adults (ages 18–35) with sentences that were either semantically meaningful or anomalous in both audio-only and audio-visual modalities in speech-shaped noise (SSN) across a range of signal-to-noise ratios. Our analyses were split into two sections: 1) keyword identification accuracy, and 2) relative visual and meaningful benefit. In our first set of analyses, we compared accuracy of keyword identification across all SNRs (0, -4, -8, -12, -16) and semantic contexts (meaningful and anomalous) in audio-only and audio-visual modalities separately. In keeping with previous studies, we predicted that young adults would achieve higher accuracy than older adults across SNRs, that audio-visual presentation would enhance accuracy relative to audio-only presentation, and that meaningful semantic context would support higher accuracy relative to an anomalous semantic context.

In our second set of analyses, we used the keyword identification accuracy results to compare the relative benefit received from visual cues and semantic cues across age groups. For these analyses, we restricted our analyses to the -8 and -12 SNRs on the basis of Ross et al. (2006), which found maximal AV benefit at -12 SNR relative to higher or lower SNRs [15]. Using intermediate SNRs (-8 and -12 SNR) circumvents ceiling or floor constraints in measuring visual benefit. We hypothesized that young adults would benefit more from visual cues in meaningful contexts relative to anomalous contexts, as was found in Van Engen, Phelps, Smiljanic, and Chandrasekaran (2014; [5]). Similarly, we predicted that young adults would use semantic cues to a greater extent in audio-visual conditions as was suggested by Van Engen et al. (2014) in their speech-shaped-noise conditions. In older adults, there are two possible outcomes for the extent to which they use visual cues in semantically meaningful relative to semantically anomalous contexts. It is possible that because older adults rely heavily on semantic context during speech perception, they will rely on visual cues more in the anomalous context relative to the meaningful context, which would be indicated by higher visual benefit scores in the anomalous context relative to the meaningful context, or no difference in visual benefit between the contexts at all. Conversely, like younger adults, older adults might use visual cues more when a semantically meaningful context is available to constrain the set of possible auditory candidates for the listener to perceive. Lastly, we expect that older adults, similar to young adults, will use semantic cues more in audio-visual conditions relative to audio-only conditions given the benefit older adults receive from audio-visual and semantically meaningful presentation, when studied independently.

## Materials and Methods

### Participants

Forty-five young adults (ages 18–35, average age = 21.8) and thirty-three older adults (ages 60–90, average age = 66.8) were recruited from the University of Texas at Austin community. All participants passed a hearing threshold test (PTA < 40 dB over 500, 1000, 2000, and 4000 Hz) and had no known psychiatric disorder. The Institutional Review Board at The University of Texas approved the experiment and materials, and written consent was obtained from each participant.

#### Neuropsychological Testing

Older adults were administered a series of standard neuropsychological tests in order to quantify their cognitive abilities. These included measures of memory (California Verbal Learning Test, CVLT; [16]), attention (Wechsler Adult Intelligence Scale, Third Edition (WAIS-III), Digit Span and Vocabulary [17]), and executive functioning (Trail Making Test A & B [18]; FAS and Wisconsin Card Sorting Task (WCST) [19]; Stroop Interference [20]). Young adults were tested with a shortened series of neuropsychological tests including the WAIS-III Digit Span, Vocabulary [17], and Stroop Interference [20].

Testing occurred during a prescreening session, and all scores were converted to age-normalized Z-scores based on each test’s standards. To ensure that our participants were within the “normal” range of neuropsychological ability, participants who scored more than two standard deviations below the mean for at least one test in each measurement group (memory, attention, and executive functioning) were not included in the study. Z-scores and demographic information are presented in Table 1 for the older adults and in Table 2 for the young adults.

**Table 1:** Neuropsychological test results and demographic information for older adults.

Measure	Mean (SD)	Range
***Neuropsychological Test***		
WAIS Vocabulary	0.86 (0.82)	-0.7 to 2.3
Digit Span	0.17 (0.79)	-1.3 to 1.7
CVLT Immediate Recall (Free)	0.83 (0.95)	-1.0 to 2.5
CVLT Delayed Recall (Free)	0.85 (0.85)	-1.0 to 2.0
CVLT Immediate Recall (Cued)	0.56 (0.86)	-1.5 to 1.5
CVLT Delayed Recall (Cued)	0.70 (0.91)	-1.0 to 2.0
CVLT Recognition False Positives	-0.50 (0.65)	-1.0 to 1.5
CVLT Recognition True Positives	-0.08 (0.99)	-3.0 to 1.0
FAS	0.13 (1.04)	-2.0 to 2.8
TMT-A	-0.44 (0.87)	-1.7 to 1.9
TMT-B	-0.42 (0.49)	-1.1 to 1.0
WCST Errors	0.44 (0.93)*	-1.4 to 2.5*
WCST Perseveration	0.50 (0.76)*	-0.9 to 2.5*
Stroop Interference	0.37 (0.88)	-1.0 to 3.0
***Demographic Information***		
Age (years)	66.82 (4.22)	60 to 75
Years of Education	15.97 (2.86)	10 to 25

* Missing one participant’s data

**Table 2:** Neuropsychological test results and demographic information for younger adults.

Measure	Mean (SD)	Range
***Neuropsychological Test***		
WAIS Vocabulary	0.84 (1.03)	-1.3 to 3.0
Digit Span	0.04 (0.92)	-1.3 to 2.3
Stroop Interference	0.76 (0.86)	-1.2 to 2.6
***Demographic Information***		
Age (years)	21.84 (3.64)	18 to 32
Years of Education	14.76 (2.29)	12 to 22

### Stimuli

#### Target Sentences

One 22-year-old native English-speaking male was video-recorded producing both semantically meaningful and semantically anomalous sentences in a sound-attenuated stage at The University of Texas at Austin. The speaker, who read the sentences from a teleprompter, was instructed to speak in a conversational manner. He was recorded using a Sony PMW-EX3 studio camera. The anomalous sentences were derived from the Syntactically Normal Sentence Test [21], and the meaningful sentences were adapted from the Basic English Lexicon [5, 22]. Both sets of sentences contained four keywords per sentence.

**Fig 1:**
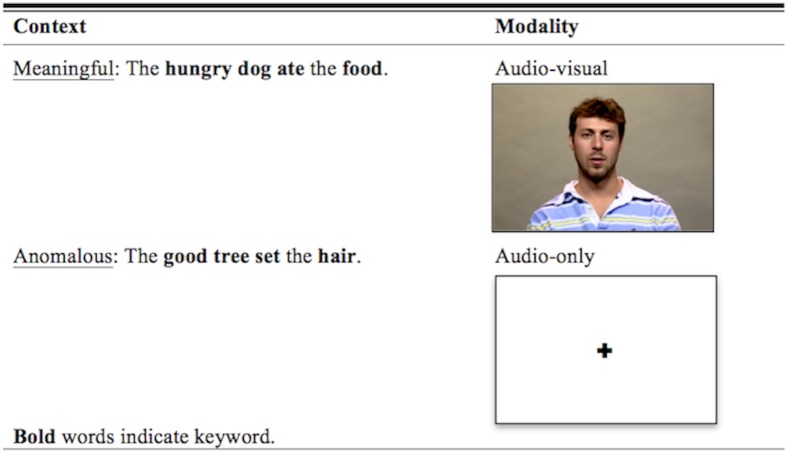
Schematic of stimuli presentation and experimental variables.

The video recording was processed through a Ross crosspoint video switcher and recorded on an AJA Pro video camera. The audio was recorded using an Audio Technica AT835b microphone at a sampling rate of 48000 Hz. The video and audio were segmented and separated using Final Cut Pro, and the audio tracks were equalized for RMS amplitude using Praat [23]. For the audio-visual stimuli, the leveled audio was reattached to the video using Final Cut Pro. Fig 1 provides a schematic of the experimental variables. The individual pictured in Fig 1 of this manuscript has given written informed consent (as outlined in PLOS ONE consent form) to publish these details.

#### Mixing noise and target sentences

The speech-shaped noise (SSN) was created by filtering white noise to match the long-term average spectrum of the 80 sentences spoken by the speaker. The RMS amplitude of the noise was scaled using Praat [23] to produce SNRs of 0, -4, -8, -12, and -16 dB when mixed with the target speech. The level of the target speech, therefore, was consistent across the experiment. In each trial, 500 ms. of noise preceded the target sentence and 500 ms. of noise followed the target sentence.

### Procedure

The speech-in-noise task was run using E-prime 2.0 in a quiet testing room. Participants viewed stimuli on a 1280 x 1024 pixel computer screen and listened to stimuli at a comfortable level through Sennheiser HD 280 Pro headphones. Participants were instructed that they would hear each target sentence preceded by one half second of noise, and that they should type the target sentence using the keyboard provided. Each trial was participant-initiated. During audio-only trials, a black fixation cross against a white background was presented on the computer monitor. During audio-visual trials, the video clip covered the entire computer screen.

Participants were presented with a total of 80 sentences across two blocks. Each block contained either meaningful sentences or anomalous sentences, and the order of these blocks was counter-balanced across participants. Within each block, participants were presented with 20 audio-only (AO) sentences and 20 audio-visual (AV) sentences with 5 levels of SNR (0, -4, -8, -12, -16). Therefore, there were 4 sentences for each context, SNR, and modality combination. Each sentence was trial-unique, that is, if a sentence were presented in the audio-only modality, it would not be presented again in the audio-visual modality. Modality and SNR were randomly presented as well. Therefore, while the presentation order of the semantic context blocks was counterbalanced between subjects, within each block every participant experienced a different order and presentation modality of a given target sentence.

Each sentence’s four keywords were hand-scored as correct or incorrect. Misspellings of the target words were acceptable as long as they did not produce another English word or change the tense or pluralization. For instance, if the target word was “plate,” “late” or “plates” would be counted as incorrect, but “platee” would be counted as correct. Because both groups’ accuracy reached floor performance (accuracy rate less than .15 correct) at -16 SNR, we excluded that condition from our analyses.

### Analyses

Results from the current study are presented in two analyses: keyword identification accuracy and relative benefit.

#### Keyword Identification Analyses

Keyword identification accuracy for every participant’s response to each sentence was analyzed using a generalized linear mixed-effects logistic model for audio-only and audio-visual modalities separately. For both analyses, accuracy on each of the four keywords per sentence served as the dependent variable in our models. Fixed effects included group (older or younger adults), context (meaningful or anomalous), SNR, and their interactions. Group and context were treated as categorical variables, and SNR, which was mean-centered, was treated as a continuous variable. The model included by-subject and by-sentence random intercepts [24]. Lastly, pure-tone averages (PTA) of each participant were added as a covariate to the model:

Response∼PTA+Group×Context×SNR+(1|Subject)+(1|Sentence) (1)

Analyses for keyword identification were performed using the *glmer* function, which fits a generalized linear mixed-effects model on independent variables that are binomial (1 or 0), and analyses for relative benefit were performed using the *lmer* function, which fits a linear mixed-effect model on independent variables that are not binomial. Both *lmer* and *glmer* are from the *lme4* package in R [25]. In addition, we utilized the *contr*.*sdif* contrast-setting function from the *MASS* package in R in order to test levels of each factor against one another [26]. To reduce the risk of over-fitting the data, we systematically removed non-significant fixed effects and their interactions, and compared the progressively simpler model to the more complex model using the likelihood ratio [27]. Only the fixed effect estimates (*β*), standard errors of the estimates (*SE*), and estimates of significance (*Z* and *p* values) from the simplest, best-fitting model are reported for the keyword identification analyses.

#### Relative Benefit Analyses

To measure the benefit each participant received from either visual cues or semantic cues, we conducted our analysis at both -12 and -8 SNR. We began at -12 SNR because it has been identified as a “sweet spot” for audiovisual integration [15]. Therefore, any differences in visual benefit due to context or group designation will likely show at this SNR. Additionally, we conducted the same analysis at -8 to test the generalizability of any pattern at an easier SNR. Similarly, we restricted our analysis of relative meaningful benefit to -12 SNR and -8 SNR.

To obtain each participant’s relative visual benefit (RVB) score for both meaningful and anomalous contexts, we used the formula:

$$RVB=\frac{AV-AO}{1-AO}$$ (2)

where *AV* represents the participant’s average accuracy during audio-visual modality presentation and *AO* represents the participant’s average accuracy during audio-only modality presentation. To investigate the effect of context and group designation on relative visual benefit score, we ran a linear mixed-effects model with relative visual benefit as the dependent variable of interest, and group and context as categorical fixed effects. We also included a by-subject random intercept.

Each participant’s relative meaningful benefit (RMB) score was derived using a similar equation to relative visual benefit:

$$RMB=\frac{MEAN-ANOM}{1-ANOM}$$ (3)

where *MEAN* is the participant’s average accuracy during semantically meaningful trials, and *ANOM* is the participant’s average accuracy during semantically anomalous trials. A mixed-effects model was carried out on RMB with group and modality as categorical fixed effects and a by-subject random intercept.

Using the relative benefit score formulas described above has the advantage of interpretation as it reflects the extent to which visual and semantic cues improve performance across a range of unimodal performance levels. The relative benefit scores also allow us to use data from a participant with floor performance in the audio-only conditions or anomalous contexts.

## Results

### Keyword Identification

#### Audio-Only Modality

**Fig 2:**
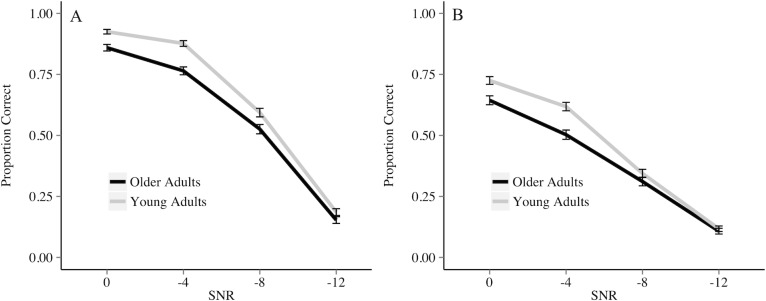
**Proportion of keywords correctly identified by older and younger adults in the audio-only meaningful (A) and anomalous (B) semantic contexts.** Bars represent standard error.

As shown in Table 3, the probability of identifying a keyword correctly in the audio-only modality was higher for the meaningful context relative to anomalous context (p < 0.0001). In addition, as the SNR became easier, the probability of identifying a keyword correctly increased (*p* < 0.0001). There is no significant effect of group or PTA. Separate models for older adults and younger adults that include audio-only accuracy as the dependent variable and PTA as the only independent variable suggest PTA significantly predicts audio-only accuracy for older adults, but not young adults. There is a significant interaction of group and SNR (*p* < 0.05), suggesting that as the SNR becomes easier, the difference between older and younger adults increases. Lastly, there is a significant interaction between context and SNR (*p* < 0.0001), suggesting that as the SNR becomes easier, the difference between meaningful and anomalous becomes larger. These results are also represented in Fig 2.

**Table 3:** Log odds of producing a correct response across group, context, and SNR in the audio-only modality.

Fixed Effect	*β*	*SE*	*Z value*	*p*
Intercept	0.61	0.31	1.95	0.05
PTA	-0.02	0.01	-1.80	0.07
Group_young adults–older adults_	0.14	0.22	0.64	0.52
Context_meaningful-anomalous_	1.39	0.07	19.28	< 0.0001
SNR	0.33	0.01	45.32	< 0.0001
Group_young adults-older adults_: SNR	0.03	0.01	2.26	< 0.05
Context_meaningful-anomalous_: SNR	0.01	0.01	9.07	< 0.0001

#### Audio-Visual Modality

**Fig 3:**
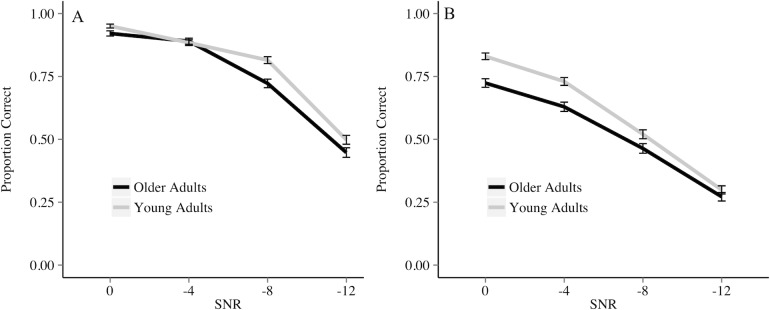
**Proportion of keywords correctly identified by older and younger adults in the audio-visual meaningful (A) and anomalous (B) semantic contexts.** Bars represent standard error.

The simplest, best fitting generalized linear mixed-effects model for the audio-visual presentation modality results included all fixed effects and their interactions. The results of the analysis are displayed in Table 4. In the audio-visual presentation modality, as pure tone averages decreased, the probability of a correct response increased (*p* < 0.01). As in the audio-only condition, separate models for older adults and younger adults that include audio-visual accuracy as the dependent variable, and PTA as the only independent variable suggest PTA significantly predicts accuracy for older adults, but not young adults. The effect of group was not significant (*p* = 0.36). The effect of context was significant (*p* < 0.0001), suggesting that a meaningful context will lead to a higher probability of keyword correctly identified. The effect of SNR was also significant (*p* < 0.0001), suggesting that as the SNR became easier, there was an increase in the probability of correctly identifying a keyword. In addition, there was a significant interaction of context and SNR (*p* < 0.0001), suggesting that as the SNR became easier, the difference between correctly identifying a keyword in the meaningful and anomalous contexts increased. Lastly there was a significant three-way interaction of group, SNR, and context suggesting that in the anomalous context but not meaningful context, as the SNR becomes easier, the difference between older adults and young adults becomes larger (anomalous: *p* < 0.05; meaningful: *p* = 0.46). These results are represented in Fig 3.

**Table 4:** Log odds of producing a correct response across group, context, and SNR in the audio-visual modality.

Fixed Effect	*β*	*SE*	*Z value*	*p*
Intercept	2.04	0.35	5.75	< 0.0001
PTA	-0.05	0.02	-3.09	< 0.01
Group_young adults–older adults_	-0.23	0.25	-0.92	0.36
Context_meaningful—anomalous_	1.55	0.08	20.21	< 0.0001
SNR	0.26	0.01	36.50	< 0.0001
Group_young adults—older adults_: Context_meaningful—anomalous_	-0.23	0.12	-1.93	0.05
Context_meaningful—anomalous_: SNR	0.09	0.01	6.34	< 0.0001
Group_young adults—older adults_: Context_meaningful_:SNR	-0.02	0.02	-0.74	0.46
Group_young adults—older adults_: Context_anomalous_:SNR	0.04	0.02	2.23	< 0.05

### Relative Benefit Analyses

#### Relative Visual Benefit

**Fig 4:**
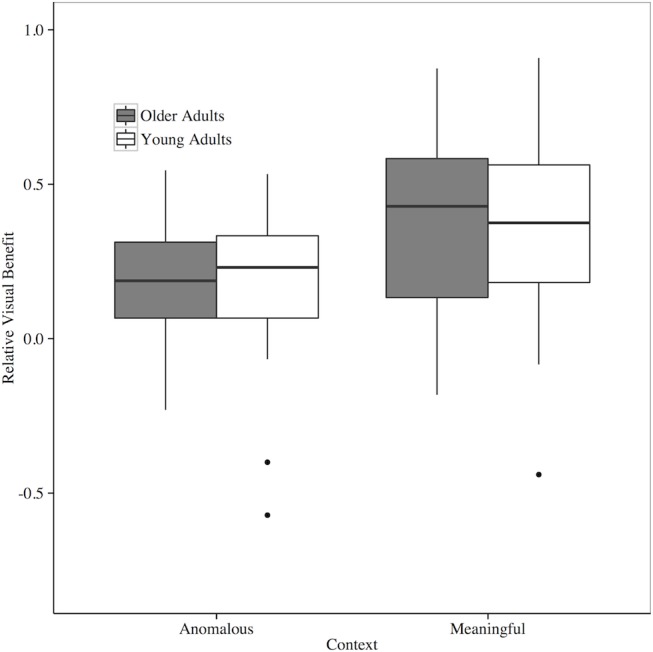
Relative visual benefit of older and younger adults in meaningful and anomalous semantic contexts at -12 SNR. Bars represent upper and lower limits of the sample data, box limits represent upper and lower quartile, and the heavy line represents the sample median.

The results of the linear mixed-effects model on the relative visual benefit in meaningful and anomalous contexts for both older and younger adults suggest no difference between older and younger adults (*β* = 0.004, *p* = 0.92), but a difference in context (*β* = 0.16, *p* < 0.0001) at -12 SNR. The positive estimate for the context fixed effect suggests that the average relative visual benefit in a meaningful context is higher than in an anomalous context. The results of the simplest, best-fitting linear mixed-effects model are presented in Table 5. Fig 4 graphically displays these results as well. Results of the linear mixed-effects model on the relative benefit in meaningful and anomalous contexts across age groups at -8 SNR suggests a similar pattern to what was found at -12 SNR. There is no difference between older and younger adults (*β* = 0.15, *p* = 0.11), but a difference in context (*β* = 0.17, *p* < 0.05) at -8 SNR. The positive estimate for the context fixed effect suggests that the average relative visual benefit in a meaningful context is higher than in an anomalous context. Lastly, we do not present relative visual benefit data from -4 and 0 SNR because we excluded numerous data points (n = 16 and n = 37, respectively) due to ceiling effects. Participants often scored perfect accuracy in the audio-only context, which placed a zero in the denominator of the RVB formula. We would also like to note that using a simple difference score (AV-AO) to measure benefit produces the same pattern of results.

**Table 5:** Results of the effect of group and semantic context on relative visual benefit at -12 SNR.

Fixed Effect	*β*	*SE*	*t value*	*p*
Intercept	0.28	0.02	13.00	< 0.0001
Group_young adults–older adults_	-0.004	0.04	-0.11	0.92
Context_meaningful–anomalous_	0.16	0.04	4.66	< 0.0001

#### Relative Meaningful Benefit

**Fig 5:**
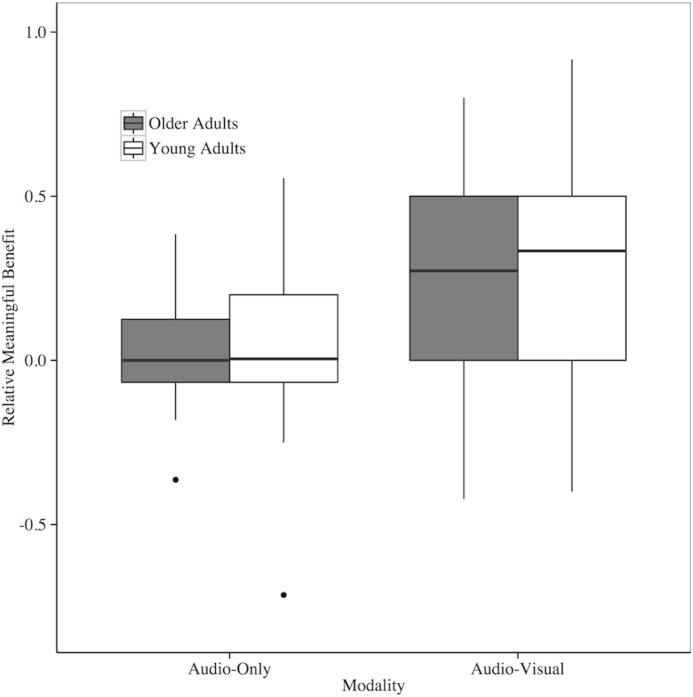
Relative meaningful benefit of younger and older adults across audio-only and audio-visual presentation modalities at -12 SNR. Bars represent upper and lower limits of the sample data, box limits represent upper and lower quartile, and the heavy line represents the sample median. Circles indicate outliers.

Results suggest that group designation is not a significant predictor of relative meaningful benefit (*β* = 0.005, *p* = 0.92), but presentation modality is (*β* = 0.20, *p* < 0.0001) at -12 SNR. The positive estimate for the modality fixed effect suggests that the average relative meaningful benefit in the audio-visual presentation modality is higher than in the audio-only presentation modality. The results of the simplest, best-fitting model are displayed in Table 6. Fig 5 displays a graphical representation of the results as well. Results at -8 SNR suggest a similar pattern of relative meaningful benefit across age groups and presentation modality as was found at -12 SNR. Group designation is not a significant predictor of relative meaningful benefit (*β* = 0.11, *p* = 0.07), but presentation modality is (*β* = 0.23, *p* < 0.0001) at -8 SNR. Lastly, we do not present relative meaningful benefit data from -4 and 0 SNR because of the high rate of a ceiling effect: 50 instances where RMB = 1.0 across presentation modalities at -4 SNR, and 81 instances where RMB = 1.0 at 0 SNR across presentation modalities.

**Table 6:** Results of the effect of group and presentation modality on relative meaningful benefit at -12 SNR.

Fixed Effect	*β*	*SE*	*t value*	*p*
Intercept	0.14	0.02	6.45	< 0.0001
Group_young adults-older adults_	0.005	0.04	0.11	0.92
Modality_audio-visual-audio only_	0.20	0.04	4.91	< 0.0001

## Discussion

The present study investigated the role of age on the ability to use visual and semantic cues to understand speech in a noisy environment. In both audio-only and audio-visual presentation modalities, as the SNR became easier, older and younger adults responded correctly more often, and the difference between the age groups in responding correctly increased, with young adults scoring higher than older adults. These results replicate prior findings suggesting that young adults have a general accuracy advantage in speech perception in speech-shaped noise relative to older adults [28]. Similarly, as the SNR became easier, the difference in accuracy between meaningful and anomalous contexts became larger, with meaningful contexts leading to a higher accuracy rate at easier SNRs. This suggests that decreasing the amount of noise present can help listeners not only by giving them more access to the speech signal, but also by allowing them to make greater use of semantic context. Only in the audio-visual presentation modality did we find a significant three-way interaction of group, context, and SNR: in the anomalous context, as the SNR became easier, the accuracy difference between young adults and older adults became larger, with young adults achieving a higher accuracy at easier SNRs. In the meaningful context, there was no change in the accuracy difference between older adults and young adults as the SNR became easier. This interaction suggests that the older adult accuracy deficit can be ameliorated to the accuracy level of young adults by the presence of both semantic context and visual cues.

One interesting pattern in the current data is that young adults outperform older adults only in the easier SNRs and in certain conditions: an anomalous context presented in the audio-visual and audio-only modalities, and a meaningful context presented in the audio-only modality. As the SNR becomes more challenging in the aforementioned conditions, the difference between groups reduces. In addition, it is worth noting that older adults maintain comparable accuracy rates to young adults in the two easiest SNRs (0 and -4) in the most supportive and commonly occurring context–audio-visual meaningful contexts. It is not until -8 SNR that older adults diverge from the young adults in terms of their speech perception ability in the audio-visual and meaningful conditions. This suggests that both visual cues and a semantic context are very important for older adults, and that with them, older adults show no deficit in speech perception ability relative to young adults.

Turning to the visual and meaningful benefit analyses, we found that older adults received the same amount of visual benefit and meaningful benefit as young adults at -12 dB and -8 dB SNRs. In addition, we found that young adults received more visual benefit during meaningful contexts relative to anomalous contexts, replicating Van Engen et al., (2014). Similarly, older adults received more visual benefit in meaningful contexts relative to anomalous contexts. In addition, young adults and older adults did not differ in the amount of meaningful benefit they received in both audio-only and audio-visual modalities. They received more meaningful benefit during audio-visual presentation relative to audio-only presentation.

To our knowledge, this study represents the first investigation of how visual and semantic cues are used by older and younger adults within the framework of a single study. Our results replicate some prior work in older and younger adults showing similar levels of visual and semantic context enhancement [3,7,10,12,13]. These results suggest that the extent to which we use visual and semantic cues remains fairly consistent as we age. It is also worth acknowledging that our older adult sample may be particularly high-functioning, and therefore may not be representative of the general population. In addition, we limited our sample to participants who passed our hearing screening of < 40 dB at several frequencies, and were above two standard deviations below the age-normalized mean of tests across memory, attention, and executive functioning. It is possible with a more heterogeneous sample in terms of hearing and neuropsychological abilities may yield larger age-related effects.

There are several limitations in the current study that should be addressed in future research. For instance, it will be important to examine visual and meaningful benefits at easier SNRs. A paradigm that equates group performance in one modality (e.g., audio-only or visual-only), for example, will circumvent the ceiling effects in accuracy (see [3]). We also acknowledge that the constrains we place on our experimental parameters, such as the pre-recorded stimuli read off of a teleprompter, may limit the generalizability of our results to real-life speech-in-noise scenarios. For instance, Gilbert et al., (2014; [29]) showed that speech spoken without the presence of noise carries different characteristics than speech spoken in the presence of noise. Therefore, since we recorded the sentences in quiet, they may differ from how they would be produced in real life. In addition, although we instructed the speaker of the stimuli to speak in a conversational manner, to mimic the temporal processing of speech in normal speech conversations, it is possible that there was still a slowing of speech given the artificial environment in which the speaker was recorded. Because older adults have difficulty with the temporal processing of speech [30], it is imperative that future work use stimuli that capture the temporal aspects of everyday speech. In addition, a further investigation of individual differences will be important to our understanding of why some young and older adults are better than others at perceiving speech, especially in noise. Anderson and colleagues (2013) provide a model for understanding how individual differences such as cognition and perceptual ability relate to speech perception in older adults; however, important cognitive measures implicated in speech perception such as processing speed [30] were not included in their analysis [31]. Therefore, future models of speech perception in the aging population should include processing speed as a predictor of speech perception in noise.

Investigating how speech production affects older adults’ use of visual and semantic cues (as in Van Engen et al., 2014) will also elucidate the extent to which older adults use other important cues in speech perception. Interestingly, Helfer (1998) found that older adults receive more visual benefit during conversational speech relative to clear speech, but the extent to which speaking style interacts with semantic benefit in older adults has not been studied. Future work should also focus on training methods that seek to enhance speech perception in older adults when visual and semantic cues are not present. Cognitive training methods, such as the one used in Anderson, White-Schwoch, Choi, and Kraus (2014; [32]) show promise of enhancing older adult speech perception under difficult listening conditions. Examining the extent to which cognitive training can improve speech perception in a variety of noise contexts carries the potential to help older adults communicate better.

In conclusion, the present study found that when both visual cues and a meaningful semantic context were present in easy SNRs, older adults showed no accuracy difference in their ability to perceive speech in noise relative to young adults. In addition, older and younger adults received the same amount of visual and meaningful benefit during speech perception, suggesting a consistency in using these cues as we age. Finally, more visual benefit was found in meaningful relative to anomalous semantic contexts and more meaningful benefit was found in the audio-visual presentation modality relative to the audio-only presentation modality. These results highlight an important facet of older adults’ speech perception: with both visual and semantic cues, older adults can excel at speech perception.
